# Pediatric emergency department visits for pedestrian injuries in relation to the enactment of Complete Streets policy

**DOI:** 10.3389/fpubh.2023.1183997

**Published:** 2023-08-21

**Authors:** Jordee M. Wells, Honggang Yi, Jingzhen Yang, Stephen J. Mooney, Alex Quistberg, Julie C. Leonard

**Affiliations:** ^1^Division of Emergency Medicine, Department of Pediatrics, Nationwide Children's Hospital, Columbus, OH, United States; ^2^Department of Biostatistics, Nanjing Medical University, Nanjing, Jiangsu, China; ^3^Center for Injury Research and Policy, The Abigail Wexner Research Institute at Nationwide Children's Hospital, Columbus, OH, United States; ^4^Department of Pediatrics, The Ohio State University College of Medicine, Columbus, OH, United States; ^5^Harborview Injury Prevention and Research Center, University of Washington, Seattle, WA, United States; ^6^Environmental and Occupational Health, Dornslife School of Public Health, Drexel University, Philadelphia, PA, United States

**Keywords:** pedestrian injury, active transport policy, Complete Streets, emergency department visits, public health

## Abstract

**Introduction:**

This study aimed to evaluate the rate of pediatric emergency department (ED) visits for pedestrian injuries in relation to the enactment of the Complete Streets policy.

**Methods:**

The National Complete Streets policies were codified by county and associated with each hospital's catchment area and date of enactment. Pedestrian injury-related ED visits were identified across 40 children's hospitals within the Pediatric Health Information System (PHIS) from 2004 to 2014. We calculated the proportion of the PHIS hospitals' catchment areas covered by any county policy. We used a generalized linear model to assess the impact of the proportion of the policy coverage on the rate of pedestrian injury-related ED visits.

**Results:**

The proportion of the population covered by Complete Streets policies increased by 23.9%, and pedestrian injury rates at PHIS hospitals decreased by 29.8% during the study period. After controlling for years, pediatric ED visits for pedestrian injuries did not change with increases in the PHIS catchment population with enacted Complete Streets policies.

**Conclusion:**

After accounting for time trends, Complete Streets policy enactment was not related to observed changes in ED visits for pedestrian injuries at PHIS hospitals.

## 1. Introduction

Pediatric pedestrian injuries comprise over half of the pediatric trauma evaluations at hospitals across the United States (1). Children are at a greater risk of morbidity and mortality from pedestrian-related injuries, but the burden of pedestrian injuries differentially impacts children within certain populations (2). The risk of child pedestrian death is greater for males, pre-school-aged children, and those in rural areas (3). Pediatric pedestrian injuries are highly impacted by the built environment as low-income areas experience high traffic burdens and interrupted sidewalks which increase the risk for potential traffic conflict (4, 5).

Complete Streets policies provide a framework for states and municipalities to develop a safe transportation system that supports active transport and accommodates all users of the road including motor vehicles, bicyclists, pedestrians, and others (6). Complete Streets emphasis on reduced traffic speeds and improved roadway design aims to increase pedestrian accessibility and decrease the likelihood of pedestrian injuries (7). Policies that influence the built environment, such as Complete Streets, require the coordinated effort of land developers, city planners, and policymakers for optimal implementation (8).

Increasing child pedestrian safety remains a top priority with the emergence of the “Vision Zero” strategy to eliminate all traffic fatalities and severe injuries while increasing safe, healthy, and equitable mobility for all (9). Complete Streets policies have demonstrated a 3-fold decrease in the number of pedestrian fatalities in states (10). While the evidence suggests that Complete Streets policies should prevent pediatric pedestrian injuries, to the best of our knowledge, no prior studies have estimated the impact of these policies on the burden of injury observed in hospitals across the country. Therefore, our study examines the relationship between the proportion of Complete Streets policy covered in a hospital catchment area and the rate of pediatric pedestrian injury-related emergency department (ED) visits.

## 2. Materials and methods

### 2.1. Study design and data sources

We conducted a retrospective cohort study on children aged 0–18 years who received care for pedestrian injuries in the EDs of the children's hospitals associated with the Pediatric Health Information System^®^ (PHIS) during 2004–2014. Hospitals included in the PHIS database represent tertiary care centers from diverse metropolitan areas across the United States. PHIS includes clinical and resource utilization data for inpatient, ambulatory surgery, ED, and observation unit patient encounters for 49 children's hospitals. PHIS captures children of all ages, races/ethnicities, and geographic regions and contains diagnoses, procedure codes, and billed transaction/utilization data.

Data on Complete Streets policies were obtained from Smart Growth America, a non-profit organization dedicated to supporting and advocating for neighborhood design and development that promote health, sustainable transportation, job growth, and community. Smart Growth America classifies policies as regulations, policies, legislation, plans, design manuals/guides, executive orders, and tax ordinances at the state, county, region, or city level. City policies were not included in this analysis. We determined hospital catchment areas in order to match them to counties and states. Hospital catchment areas were determined by looking up catchment information for each hospital in the database on the hospital's website or other resources. While some hospitals may cover only small areas or share areas with other children's hospitals, some may cover multiple counties or states; thus, the population they serve may have had varying Complete Streets population. To address this, we calculated the proportion of the population within PHIS hospitals' catchment areas covered by Complete Streets policies between 2004 and 2014. We considered a Complete Street policy to have started for analytical purposes in the year after its adoption. The presence of any of these policies during a given year qualified as a proportion of the population covered by the policy at the state or county level, respectively.

### 2.2. Study population

#### 2.2.1. Inclusion criteria

From all ED visit records in PHIS, children aged 0 to 18 years with corresponding ICD-9-CM pedestrian external causes of injury codes (e-codes) that ranged from E800.2 to E828.0 were included. Examples of the corresponding injuries include railway collision-injuring pedestrian, motor vehicle traffic collision-injuring pedestrian, motor vehicle non-traffic collision-injuring pedestrian, pedal cycle collision injuring-pedestrian, and animal-drawn vehicle collision-injuring pedestrian.

#### 2.2.2. Exclusion criteria

To ensure data quality, we assessed the percentages of all injury visits with a reported e-code at a given hospital by year. We excluded data from hospital years that had <85% of injury visits with one or more e-code because if >15% of data are missing, it is likely that the data are not missing by chance and are sufficient to risk biasing results (11).

### 2.3. Variables

#### 2.3.1. Exposure variables

We created two policy variables for this study that reflect the coverage of Complete Streets policies within each hospital's respective catchment areas: (1) policy coverage at the county level and (2) policy coverage at either the state or county level. Complete Streets policies can be enacted at either the state or county level as legislation resolutions or executive orders. The distinction between policy at the county level and state level is reflective of policy enactment. Hospital catchment areas might cover multiple counties and, in some circumstances, states.

The proportion of the youth population covered by the policy, the main exposure variable of interest, was calculated as the estimated number of youth aged 0–18 years within a hospital's catchment area that was covered by the Complete Streets policy at the county and/or state level at a given year divided by the total number of youth aged 0–18 years within the same hospital's catchment area during the same time period. We used 1-year American Community Survey estimates to estimate the youth population aged 0–18 years corresponding to each catchment area in a given year. If a county was covered by multiple hospitals (i.e., intersecting catchment areas), the shared coverage county was included by each of these hospitals in the calculation (i.e., the county was included in the calculation for each hospital).

#### 2.3.2. Outcome variables

The rate of pedestrian injury-related ED visits was calculated as the number of pedestrian injury-related ED visits in a specific hospital in a specific year divided by the total number of injury-related ED visits in the same hospital during the same year and then multiplied by 10,000.

### 2.4. Data analysis

We described the patient and injury characteristics of children aged 0 to 18 years with pedestrian external causes of injury who visited pediatric EDs in PHIS hospitals between 2004 and 2014. We calculated the proportion of the population within PHIS hospitals' catchment areas covered by the Complete Streets policy at the county or state level from 2004 to 2014, as well as the rate of pedestrian injury-related ED visits per 10,000 injury-related ED visits, along with 95% confidence intervals over time for children aged 0–18 years from 2004 to 2014. To measure the impact of the Complete Streets policy, we used the proportion of the youth population covered by the Complete Streets policy at the county and/or state level as an exposure of interest. If the proportion of coverage was 0, the Complete Streets policy was not enacted.

We used a generalized linear model (GLM) to evaluate the impact of the Complete Streets policy on the rates of pedestrian injury-related ED visits per 10,000 injury-related ED visits, with Poisson distribution and log link function. The offset was the total number of injury-related ED visits. The rate ratio was calculated using every 1% increase in policy coverage (at county and/or state level, depending on the analysis)-associated rate of pedestrian injury-related ED visits per 10,000 injury-related ED visits. A generalized estimation equation (GEE) method with a working exchangeable correlation structure was specified in the GLM to account for within-hospital correlations (e.g., in coding practices) over time. To account for statistical biases caused by year differences, we adjusted for the study year in our GLM. A *p* ≤ 0.05 was considered to be statistically significant.

## 3. Results

**Figure 1:**
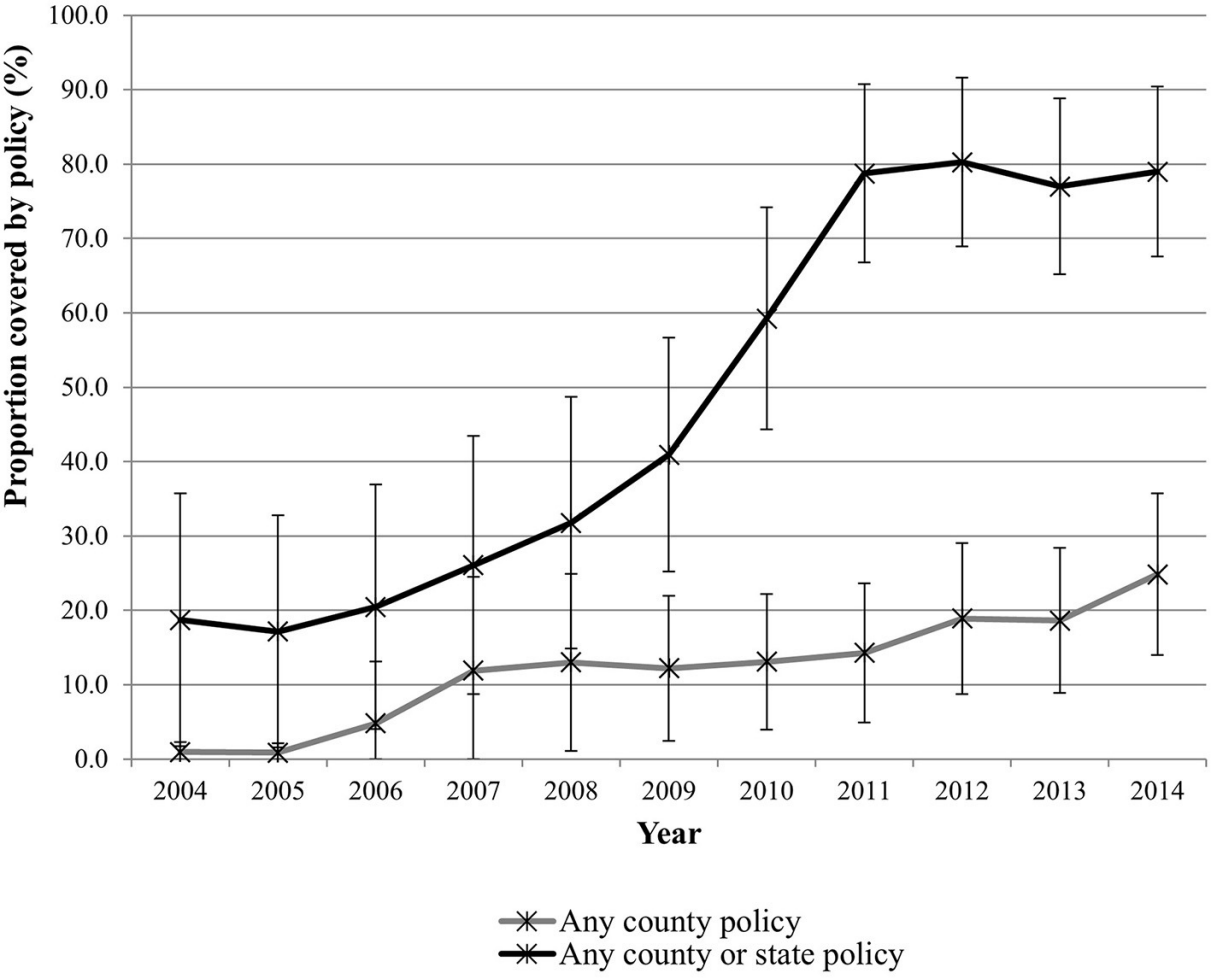
Proportion of the population within PHIS hospitals' catchment covered by Complete Streets policies.

We identified 29,300 ED visits for children with pedestrian-related injuries from 2004 to 2014 that met our inclusion criteria (763 children who were transferred to another acute care facility were excluded to avoid double counting). Table 1 presents the characteristics of the study population. Nearly two-thirds of pedestrian-related visits were for children between the ages of 5 and 9 years (30.4%) and 10 and 14 years (32.7%). In total, 62% of the injured were male. The most common primary insurer was Medicaid/Medicare (56.2%), followed by private insurance (21.8%). As expected, most injured children were from urban/suburban areas (91.6%). Most children with pedestrian injuries were discharged home from the ED following care (95.6%). Of the ED visits that led to inpatient hospitalization, 2,874 required the intensive care unit, 3,790 went to the operating room, and 1,578 required mechanical ventilation. Less than 2% of children died due to their injuries in the hospital. Complete Streets policy coverage in the catchment areas increased over time at both the county and state levels (Figure 1).

**Table 1:** Patients' characteristics.

**Characteristics**	**No. (%)**
**Age group**	
< 5	6,717 (22.4)
5–9	9,102 (30.4)
10–14	9,783 (32.7)
15–18	4,336 (14.5)
**Gender**	
Male	18,547 (62.0)
Female	11,317 (37.8)
**Race**	
White	11,806 (39.4)
Black	12,127 (40.5)
Other^a^	6,005 (20.1)
**Ethnicity**	
Hispanic or Latino	6,202 (20.7)
Not Hispanic or Latino	14,637 (48.9)
Other^b^	9,099 (30.4)
**Primary payment**	
Private	6,529 (21.8)
Medicaid/Medicare/Other public	16,795 (56.1)
Uninsured	2,320 (7.7)
Other/Unknown	4,294 (14.3)
**Disposition**	
Discharged/transferred to home health services	288 (1.0)
Discharged/transferred to home or self-care	28,617 (95.6)
Left against medical advice (AMA)	30 (0.1)
Expired	303 (1.0)
Other	700 (2.3)
**Area**	
Urban/suburban	27,434 (91.6)
Not urban	1,692 (5.7)
Unknown	812 (2.7)
**Using ICU**	
No	27,064 (90.4)
Yes	2,874 (9.6)
**Operating room charge**	
No	26,148 (87.3)
Yes	3,790 (12.7)
**Mechanical ventilation**	
No	28,360 (94.7)
Yes	1,578 (5.3)
No	29,537 (98.7)
Yes	401 (1.3)
**Patients type**	
Directly admitted	2,694 (9.0)
ED to inpatient	7,812 (26.1)
ED only	19,432 (64.9)

Total percentage of <100 is due to missing value (s).

a other includes native Hawaiians, other Pacific Islanders, Samoan, American Indians, Asian, Alaska Natives or Unknown.

b other includes multiethnic, other ethnicity and unknown.

**Figure 2:**
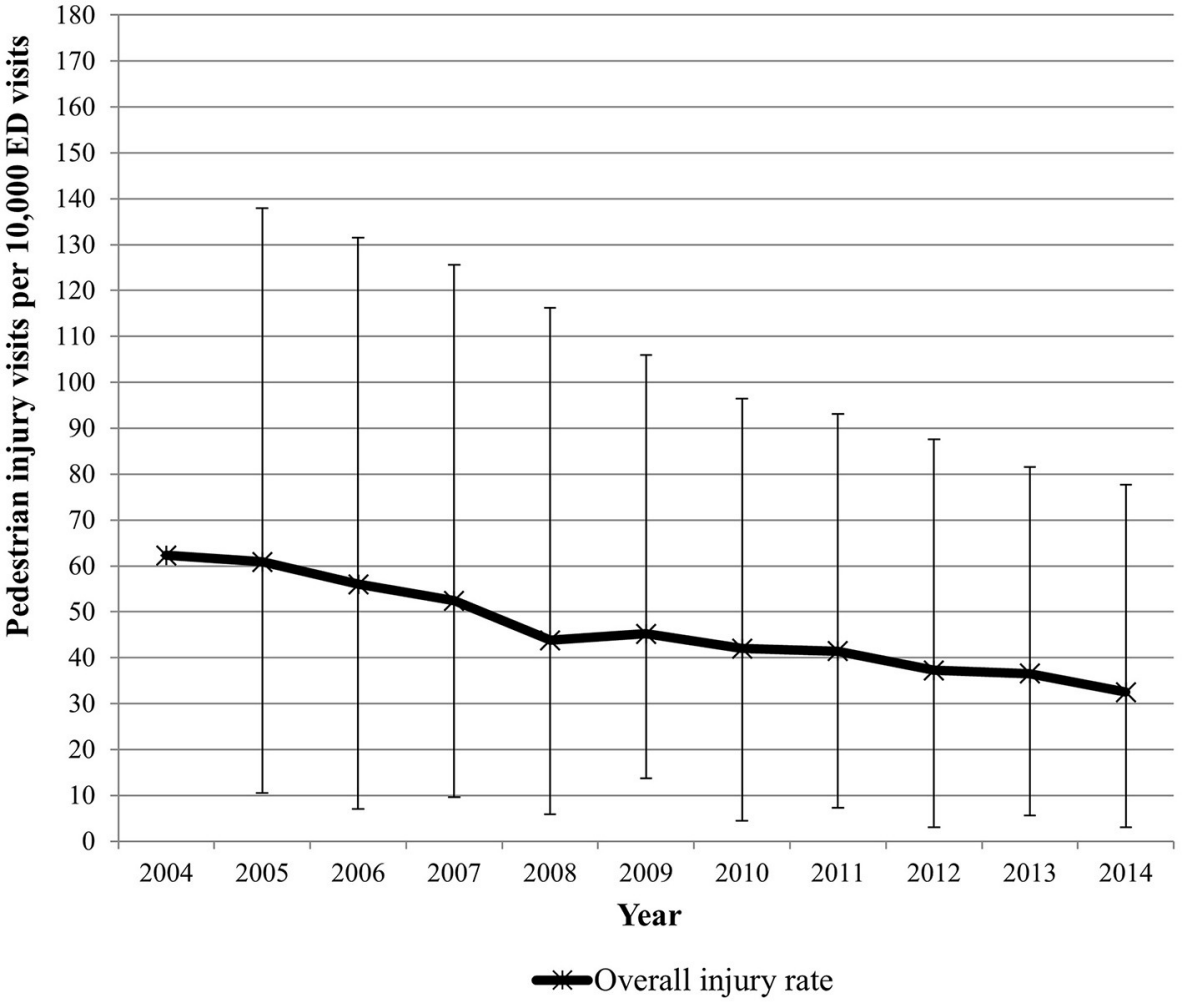
Pedestrian injury rates by year.

The proportion of youth in PHIS hospitals' catchment areas that were covered by at least **one** Complete Streets policy at the county level increased from 0.92% to 24.84% over the study period. The proportion of youth covered by at least **one** Complete Streets policy at the county or state level increased from 18.69% to 78.98% over the study period. The rate of pedestrian injury-related ED visits declined from 62.3 per 10,000 visits in 2004 to 32.5 per 10,000 visits in 2014 (Figure 2).

Multivariable analyses evaluated the association between the proportion of youth covered by Complete Streets policies and the pedestrian injury rates (Table 2). The results from the unadjusted analysis showed that as the proportion of the youth population covered by any county Complete Streets policy increased by 1%, the rate of pedestrian injury-related ED visits proportionally decreased by 0.34%. However, after adjusting for the year of ED visits, the proportion of the youth population covered by Complete Streets policies was not associated with pedestrian injury-related ED visits. The adjusted analyses of any county-level or state-level Complete Streets policy showed comparable findings (Table 2).

**Table 2:** Proportion of persons covered by the complete street policy and rate of pedestrian injury per 10,000 ED visits.

**Variable^b^**	**Rate of pedestrian injury (per 10,000 ED injury visits)** ^**a**^							
	**Un-adjusted**				**Adjusted** ^c^			
	***B***	***SE***	***P***	***IRR** ^*d*^*	***B***	***SE***	***P***	***IRR** ^*d*^*
Intercept	−5.7604	0.1797	< 0.0001		129.0584	20.7183	< 0.0001	
Proportion of persons covered by any county policy (%)	−0.0034	0.0016	0.0350	0.9967 (0.9935–0.9998)	0.0015	0.0016	0.3562	1.0015 (0.9983–1.0046)
Intercept	−5.4587	0.1523	< 0.0001		129.1573	23.8832	< 0.0001	
Proportion of persons covered by any policy (%)	−0.0032	0.0009	0.0001	0.9968 (0.9951–0.9984)	0.0004	0.0009	0.6697	1.0004 (0.9986–1.0022)

a generalized linear model (GLM) was used, with Poisson distribution and the log link function. The offset is the total number of ED visits. Generalized estimation equation (GEE) method with a working exchangeable correlation structure was specified in the GLM to account for correlations of the pedestrian injuries treated in the same hospital across the study period.

b each predictor variable was proportion of person covered by policy (%) and included in a separated GLM as a continuous variable.

c adjusted by study year.

d rate of pedestrian injury per 10,000 ED visits of in hospitals without policy coverage was a reference.

## 4. Discussion

We used the PHIS database to assess ED visits for both fatal and non-fatal pediatric pedestrian injuries in relation to the enactment of Complete Streets policies in multiple regions across the country. Complete Streets policy coverage in the PHIS catchment areas increased over time at both the county and state levels, and over the same time period, the rate of pedestrian injury-related ED visits declined. However, we did not find evidence that the enactment of these active transport policies contributed directly to the observed decline in pedestrian injury-related ED visits.

While we observed a decline in pedestrian-related ED visits over the period that Complete Streets policies were enacted, our data also indicate that pediatric pedestrian injuries were on the decline prior to the enactment of these policies. We recognize that the proportion of pediatric pedestrian injuries may be declining due to an increase in total pediatric injuries that are present in children's hospitals. According to the National Highway Traffic Safety Administration, trends in traffic fatalities have been decreasing for over **four** decades and most recently represent a fatality rate of 1.16 per 100 million vehicle miles traveled in 2017 (12). This trend may be explained by secular trends. Roadway size and structure were shown to be positive mitigating strategies in urban planning to improve traffic safety and lower fatality rates (13). Improvements in vehicular safety features over time have reduced the number of fatalities in the United States and likely contribute to the observed decline (14).

Transport engineering has the potential to improve health by decreasing pedestrian injuries and promoting active transport (15). A rise in pediatric pedestrian injuries may be the tradeoff for increased pedestrian activity within communities following the enactment of Complete Streets policies. Some studies have similarly found that policies promoting active transport can increase the total number of injuries due to increased community participation. An audit of an urban corridor with Complete Streets intersections demonstrated greater volumes of pedestrian activity with increased opportunity for potential injury (16). Applying G-computation to evaluate the effects of Complete Streets policies on the size of the cycling population on adult bicyclists fatalities demonstrated that the change in the denominator can make a decrease in the numerator, looking like a null effect (17). The lack of surrogate measures within communities for engagement in active transport activities for children, such as walking and bicycling, represents a gap in available injury research resources. Future efforts should be made to incorporate these measures in broad public surveys such as the United States Census, which will allow for better policy evaluation.

Studies have examined the conditions under which the risk of pedestrian fatalities is the highest to better inform transport engineering efforts (18). Additionally, studies have assessed the impact of motor vehicle crash avoidance technology including pedestrian detection on pedestrian fatalities (19). However, lessons from the Heinrich pyramid suggest that fatal injuries are just the tip of the overall burden of traffic-related collisions (20). If there is continued reliance on pedestrian fatalities as the outcome, then the potential effects of policy on non-fatal pedestrian injuries will be underreported. Our study contributes to the literature by evaluating the larger population of ED visits for pediatric pedestrian injuries as fatalities represented **<**2% of the ED visits. Improvements in ED care may prevent fatalities over time; thus, examining non-fatal injuries gives us a better view of the impacts of policies in preventing collisions.

We highlight that not all motor vehicle-pedestrian collisions result in ED visits. Our study thus did not include collisions resulting in minor injuries that did not require medical evaluation, or collisions resulting in injuries that were evaluated in ambulatory settings including primary care and urgent care. Whereas full evaluation of policy changes should account for such collisions, our study focuses on more severe injuries, which account for most of the collision health burden.

There are several limitations to this study that may impact our findings. Most importantly, no measure of pediatric pedestrian activity was available to use in our analysis. Thus, it is possible that the observed unchanged injury rate could result from a combination of reduced per-pedestrian injury risk due to the improved walking environment combined with an increased number of pedestrians at risk due to those same improvements in the walking environment. As our unit of policy analysis is a county, we recognize that there are differences in high-risk and low-risk areas within a county or neighborhoods for pedestrian road injuries that were not addressed. The data obtained from PHIS were reflective of only ED visits at children's hospitals; therefore, pediatric pedestrian injuries observed at other emergency departments or in other care settings were not included. However, we are aware of no reason to believe changes in ED choice for injured child pedestrians would be correlated with Complete Streets policy enactment, so we do not anticipate biases resulting from this incomplete coverage. However, during the study period, national trauma policy transitioned to encourage pediatric patients to be directed to tertiary care centers as opposed to community hospitals, and this may have affected our observed injury rates.

## 5. Conclusion

Complete Streets policy enactment increased over time, and the rates of ED visits for pediatric pedestrian injuries decreased. However, after controlling for year, Complete Streets policy enactment was not associated with the rate of pediatric pedestrian injury-related ED visits. This may be due to increased pedestrian activity in areas covered by these policies or other unmeasured factors such as changes in city ordinances. Further research is necessary to fully understand the effects of road safety policies on walking and pedestrian injuries among children.

## Data availability statement

The raw data supporting the conclusions of this article will be made available by the authors, without undue reservation.

## Author contributions

JY, SM, AQ, and JL conceptualized and designed the study, obtained the data, contributed to data analysis and interpretation, drafted and revised the manuscript, and approved the final manuscript while agreeing to be accountable for all aspects of the study. JW contributed to data analysis and interpretation, drafted and revised the manuscript, and approved the final manuscript while agreeing to be accountable for all aspects of the study. HY obtained the data, contributed to data analysis and interpretation, drafted and revised the manuscript, and approved the final manuscript while agreeing to be accountable for all aspects of the study.
